# A huge metaplastic breast carcinoma with chest wall invasion and pulmonary metastasis: a rare case report

**DOI:** 10.1093/omcr/omac027

**Published:** 2022-03-16

**Authors:** Haidara Kherbek, Ismaeel Deeb, Haya Ali, Mais Halloum, Zuheir Alshehabi, Wassim Ali

**Affiliations:** Faculty of Medicine, Tishreen University, Latakia, Syria; Faculty of Medicine, Tishreen University, Latakia, Syria; Faculty of Medicine, Tishreen University, Latakia, Syria; Faculty of Medicine, Tishreen University, Latakia, Syria; Department of Pathology, Cancer Research Center, Tishreen University, Latakia, Syria; Department of Thoracic Surgery, Tishreen University Hospital, Latakia, Syria

## Abstract

Metaplastic breast carcinoma (MBC) is a diverse group of invasive breast carcinoma, in which the neoplastic epithelium differentiates toward squamous cells or mesenchymal looking elements, including but not restricted to spindle, osseous and chondroid cells. MBC was formally considered a distinct pathological pattern by WHO classification of breast tumors in 2000. We report the case of a 49-year-old Syrian female who presented to our hospital due to a painful huge mass in her right breast. Radiographic and clinical findings were highly indicative of breast carcinoma. Therefore, a core needle biopsy was performed, and surprisingly, microscopic examination suggested the diagnosis of soft tissue sarcoma, whereas immune stains confirmed the diagnosis of metaplastic carcinoma. We aim to introduce a challenging case that clarifies the rarity of this tumor, and the methods we used in diagnosing, examining and treating this malignancy.

## INTRODUCTION

Metaplastic breast carcinoma (MBC) represents an extremely rare entity, accounting for <1% of all breast cancers [1]. It is formed of various cell types including: spindle cells that are the most common type, in addition to squamous and/or mesenchymal cell types [2]. Most MBCs are triple negative for estrogen (ER), progesterone (PR) and HER-2 receptors indicating a poor prognosis [3]. The information about tumor presentation, features, prognosis and treatment are limited because it was not formally recognized as a distinct pathological pattern by the World Health Organization (WHO) until 2000 [4]. In this article, we introduce a challenging case that clarifies the characteristics of this malignancy and the methods we used in diagnosing, examining and treating it.

## CASE REPORT

**Figure 1:**
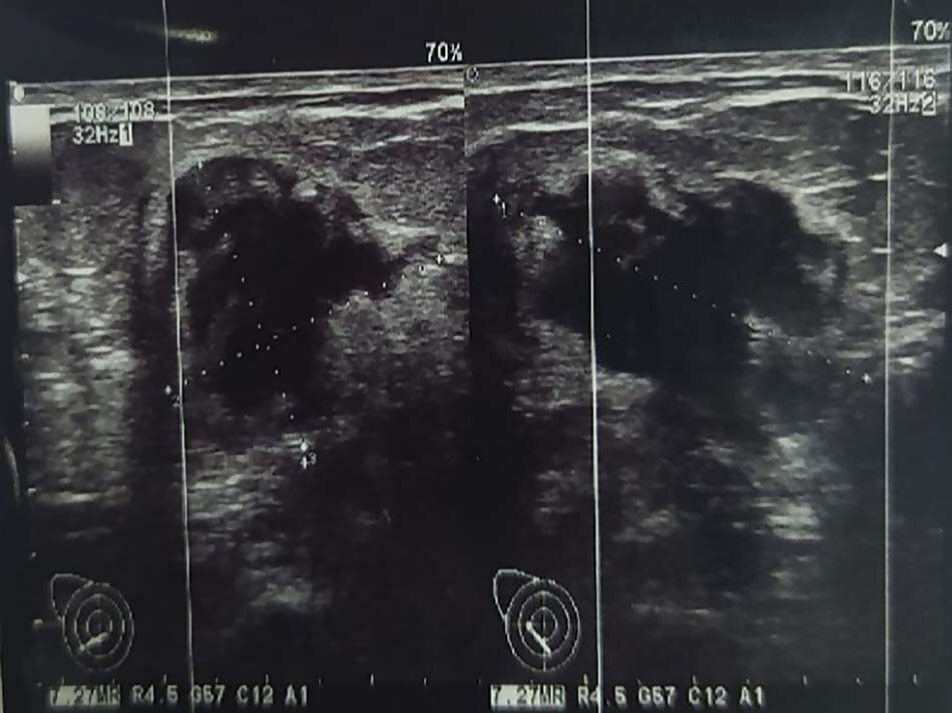
Breast US shows a hypoechoic lobulated mass with heterogeneous content, measuring 14 cm in diameter.

**Figure 2:**
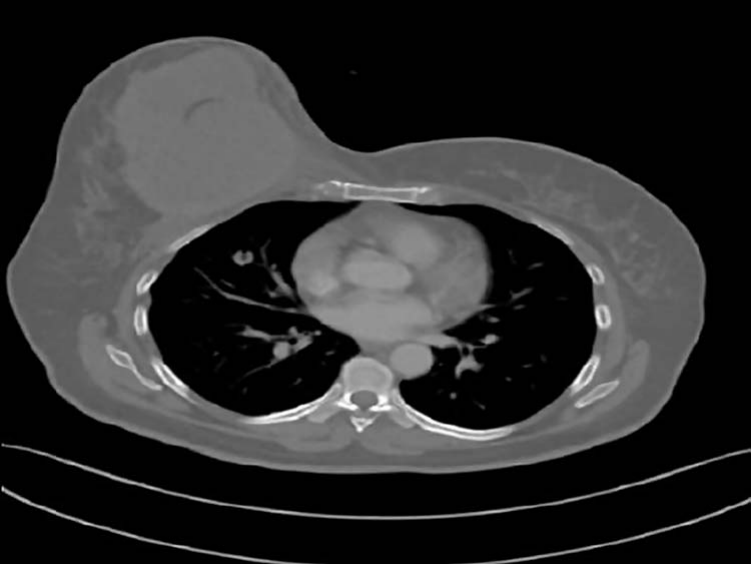
Chest CT shows a high-density mass in the right breast infiltrating the chest wall, accompanied with a metastatic lesion in the right lung.

**Figure 3:**
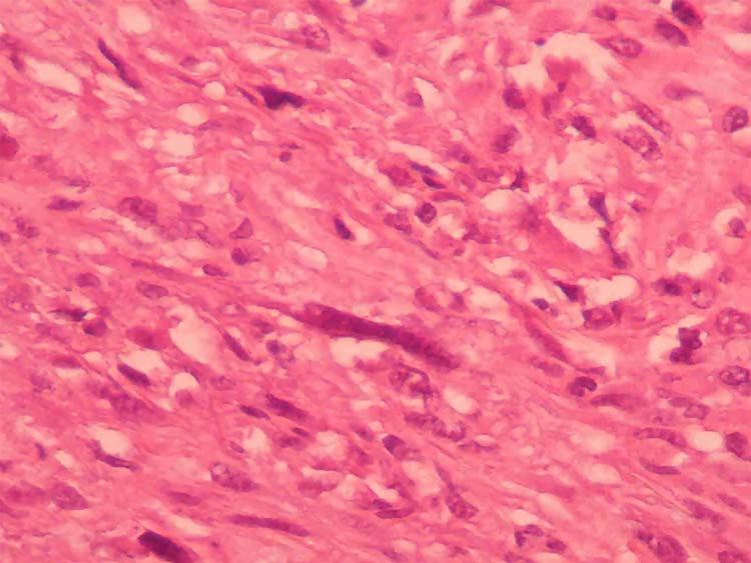
Tumor nests interspersed with chondromyxoid matrix (hematoxylin and eosin [H&E] ×400).

**Figure 4:**
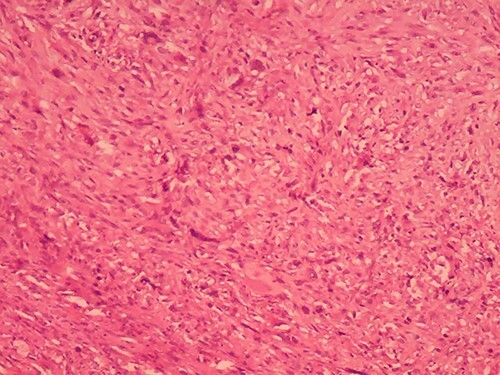
Metaplastic carcinoma composed of high-grade spindle cells with angulated hyperchromatic nuclei in a collagenous stroma. (H&E ×100).

**Figure 5:**
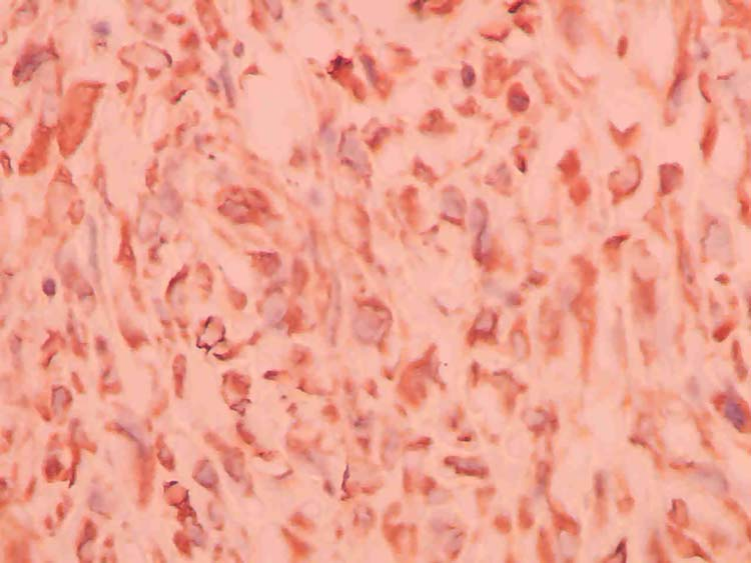
Immunohistochemistry of the tumor shows positivity for vimentin.

**Figure 6:**
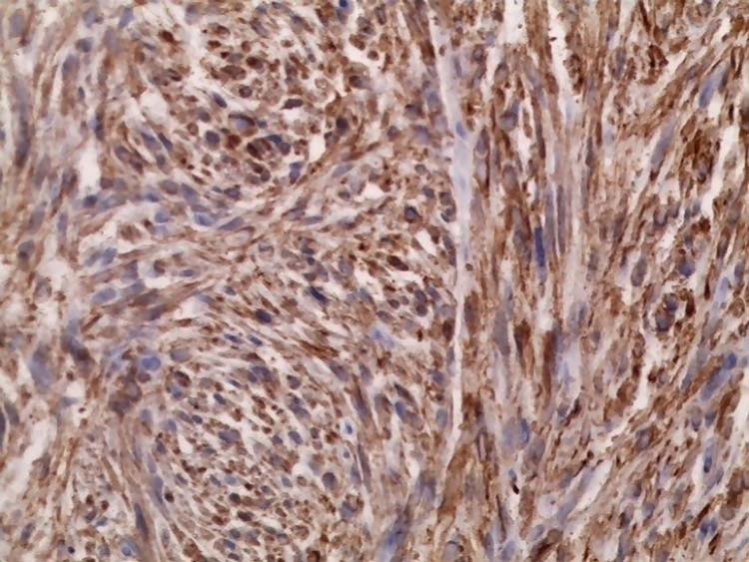
Immunohistochemistry of the tumor shows positivity for P63.

**Figure 7:**
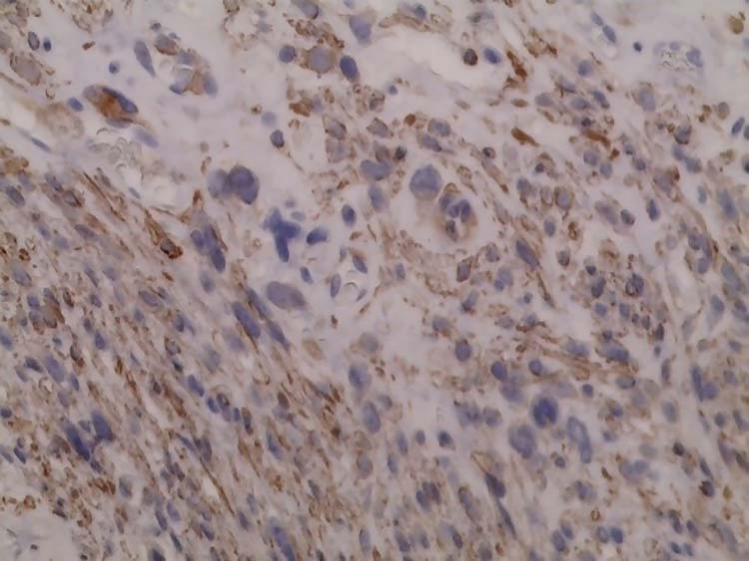
Immunohistochemistry of the tumor shows positivity for CK 5/6.

**Figure 8:**
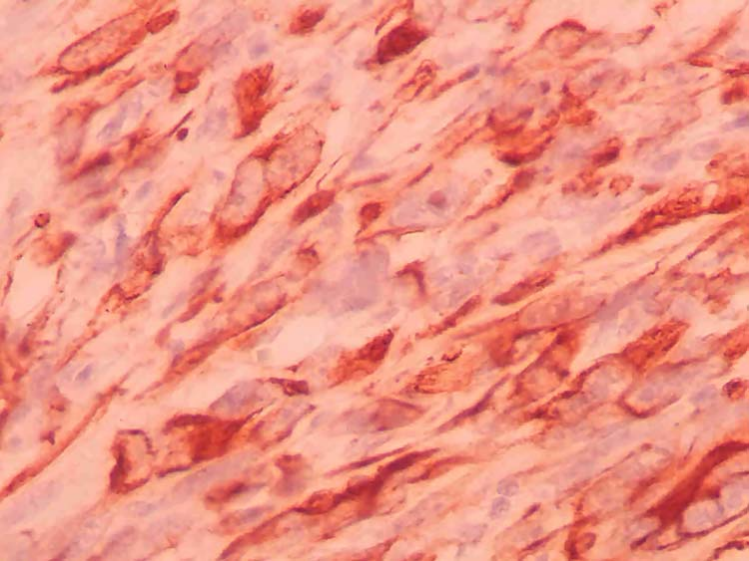
Immunohistochemistry of the tumor shows positivity for pancytokeratin.

**Figure 9:**
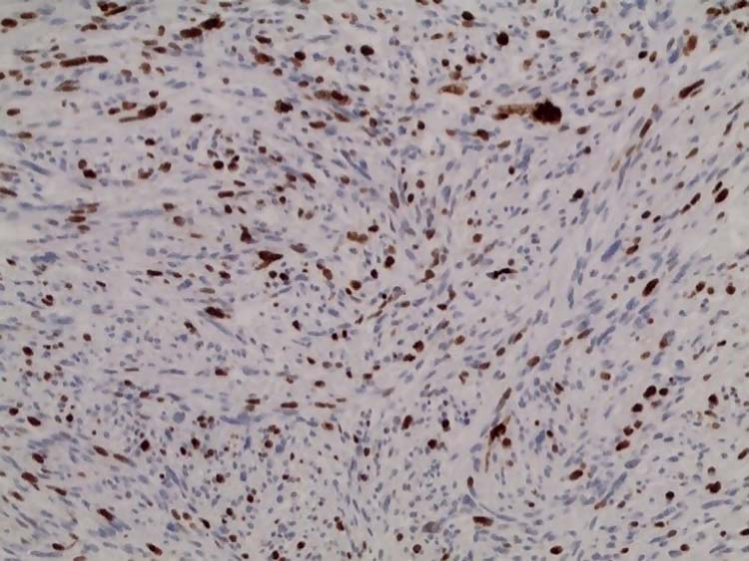
Immunohistochemistry of the tumor shows positivity for Ki67: high rate ~ 30%.

We report the case of a 49-year-old Syrian female, who was admitted to our hospital due to a painful huge lump in her right breast that had rapidly enlarged in the last 2 months without any signs of skin changes or nipple discharge. The patient denied any history of trauma, but she underwent a surgery to remove a benign ovarian cyst 15 years ago. In addition, her medical and family history were insignificant. Physical examination revealed a painful firm mass in the medial half of her right breast with painful nodular formations beside the areola and in the right axilla. Laboratory findings showed elevated C-reactive protein (CRP), whereas all other results were normal. Breast ultrasonography (US) (Fig. 1) showed a hypoechoic lobulated mass with heterogeneous content measuring 14 cm in diameter, localized in the medial half of the right breast, along with another nodular formation near the areola and two enlarged lymph nodes at the right axillary region. Pleural US revealed mild bilateral pleural effusion. Abdominal US was normal. Chest computed tomography (CT) (Fig. 2) showed a tumor infiltrating the chest wall with a metastatic lesion in her right lung, whereas abdominal and pelvic CTs were normal. Consequently, radiographic and clinical findings were highly suggestive of breast carcinoma, which was our primary differential diagnosis. Therefore, the patient underwent a core needle biopsy, which revealed a specimen consists of seven soft to rubbery white-yellowish, ivory-white cores of tissue ranging from 6 to 15 mm length. Microscopic exam revealed heterologous epithelioid and spindle components including myxoid, chondroid, osseous and fibrous differentiations (Fig. 3), with a wide range of atypia, extending from minimal atypia to frankly malignant. In addition to high-grade spindle cell lesion (Fig. 4) with pleomorphic nuclei and occasional mitotic figures, including atypical mitoses, there were also foci of necrosis. These findings correlate with breast sarcoma, but immunohistochemical (IHC) staining was positive for vimentin (VIM) (Fig. 5), P63 (Fig. 6), CK5/6 (Fig. 7) and pancytokeratin (Pan-CK) (Fig. 8), whereas ER, PR and HER2 were all negative, Ki67 ~ 30% (Fig. 9), which in turn, ruled out the breast sarcoma and confirmed the diagnosis of MBC. Clinical TNM stage of T4N1M1 was given. Based on the radiological and clinical findings, as well as the advanced tumor stage, which all indicate a poor prognosis, radical mastectomy and chemotherapy of cyclophosphamide and doxorubicin were performed as a palliative treatment. Then the patient will be monitored at periodic intervals of 3 months for the first 2 years.

## DISCUSSION

MBC is a very rare malignancy, which consists of a variety of cell types, mainly spindle cells in addition to squamous and/or mesenchymal cell types [2], accounting for <1% of all breast tumors [1]. It has a unique IHC pattern includes positivity of VIM and Pan-CK and negativity of ER, PR and Her2neu [5]. The 2011 WHO classified MBC into the following subtypes: low-grade adeno-squamous carcinoma, squamous cell carcinoma, MBC with mesenchymal differentiation, spindle cell carcinoma and fibromatosis-like metaplastic carcinoma, noticing that a large portion of MBCs reveal a mixture of different elements [6]. A cohort study showed that the most common subtype was matrix-producing carcinoma, followed by squamous and spindle cell carcinomas, respectively [7]. Most cases present with rapidly growing masses >2 cm [1] and very few ones present with axillary lymph nodes metastases with an incidence ranging from 6 to 26% [3]. Most cases had patients with a median age extending from 48 to 59 years old [8], with a median size of 3 cm [7]. Studies revealed that the accurate diagnosis of MBC is low [3]. So that, patients with MBCs tend to be diagnosed with a higher tumor stage except for fibromatosis-like metaplastic carcinoma and low-grade adenosquamous carcinoma, which are usually detected at early stages [7]. Sonography demonstrates a heterogeneous, hypoechoic solid mass or a mass with a mixture of cystic and solid compounds as seen in our patient [8]. Histologically, the primary differential diagnosis includes myoepithelial carcinoma, myofibroblastic tumors, malignant phyllodes tumors, pleomorphic adenoma and adenomyoepithelioma. In contrast with MBCs subtypes with squamous differentiation, mesenchymal subtypes require IHC staining to be differentiated from breast sarcoma. The mesenchymal subtypes have co-expression of mesenchymal (VIM) and epithelial cell (CK) markers, which distinguish it from breast sarcoma [9]. Furthermore, MBCs show more benign features comparing to invasive ductal carcinoma [9]. We should keep in mind that it is essential to differentiate MBC from phyllodes tumor, which has a leaf-like growth pattern in addition to negativity of CK [10]. The main treatment of MBC is surgery followed by adjuvant radiotherapy [3] and despite being a chemo-resistant tumor, chemotherapy is administered in 53.4–73.1% of cases [7]. In conclusion, our case revealed a tumor characterized by a large size on presentation, which rapidly enlarged in a short period of time, accompanying with metastatic lesions seen in the nearby structures such as the lung and the axillary lymph nodes, as well as a triple negative pattern and a sarcoma-like pattern on microscopic exam; leading to sub-optimal response to systemic therapies. On the other hand, palliative treatment was performed due to the very poor prognosis.
